# A new species of *Neopanorpa* with an extremely long notal organ from Sichuan, China (Mecoptera, Panorpidae)

**DOI:** 10.3897/zookeys.750.23486

**Published:** 2018-04-18

**Authors:** Meng Wang, Bao-Zhen Hua

**Affiliations:** 1 Key Laboratory of Plant Protection Resources and Pest Management, Ministry of Education, Entomological Museum, Northwest A&F University, Yangling, Shaanxi 712100, China

**Keywords:** Gonostylus, Insecta, mating behavior, postnotal organ

## Abstract

*Neopanorpa setigera* **sp. n.** is described and illustrated from Sichuan, China. It is characterized by an extremely long notal process, a well-developed postnotal organ, and a pair of setiferous gonostyli. This is the second species of *Neopanorpa* van der Weele with an extremely long notal organ in Sichuan. A key to Sichuan species of *Neopanorpa* is provided.

## Introduction

Panorpidae are the most speciose family of Mecoptera with approximately 420 species assigned in seven genera (Hu et al. 2015; Gao et al. 2016). They are commonly called scorpionflies due to their swollen male genital bulb bearing striking resemblance to the sting of scorpions. Scorpionflies are generally distributed over the Holarctic and the Oriental regions. They often inhabit deep and mesic forests and are commonly encountered among dense herbaceous vegetation in lowlands or open areas near water (Carpenter 1931a; Byers and Thornhill 1983). They are saprophagous insects, mainly feeding on dead arthropods (Byers and Thornhill 1983). They also feed on fruits, and likely flower pollens sometimes (Byers and Thornhill 1983).

Panorpidae exhibit diversity in mating behavior and strategies. The males generally provide a prey or salivary secretions as nuptial gifts, or practice coercive mating to the female during copulation (Thornhill 1980; Zhong and Hua 2013; Zhong et al. 2015a, b). Recent studies show that the mating strategies are closely related with some non-genital structures, for example the notal organ and anal horns (Zhong and Hua 2013; Zhong et al. 2015a). The notal organ is the posterior median process of abdominal tergum III (notal process) paired with the minor projection on tergum IV (postnotal organ) as an appendage to clamp the wings of the female during copulation and plays a significant role in prolonging the copulatory duration (Mickoleit 1971; Thornhill 1980; Thornhill and Sauer 1991; Zhong and Hua 2013). In *Furcatopanorpa longihypovalva* (Hua & Cai) the absence of notal organ leads it to prolong the copulatory duration by providing serial salivary secretions instead of seizing the female wings (Zhong et al. 2015b). In contrast, *Neopanorpa longiprocessa* Hua & Chou bears a greatly elongate notal organ, and lacks nuptial feeding during courtship and copulation (Zhong and Hua 2013).

*Neopanorpa* van der Weele is the second largest genus of Panorpidae endemic to the Oriental Region. China owns the greatest species richness with 95 of the total 160 species to date (Wang and Hua 2017). The genus is characterized by vein 1A ending before the origin of Rs. Most species are morphologically distinguished from other genera by the slender wings, an elongate notal organ, and undeveloped salivary glands (Ma et al. 2011). *Neopanorpa* eggs exhibit separate and independent neighbouring ridges on the exochorion, whereas those ridges are fused together among other genera in Panorpidae (Ma et al. 2009). In addition, the larvae adopt a euedaphic life with the presence of shallow furrows on the head and prolegs of the first four abdominal segments greatly reduced (Jiang and Hua 2015).

Sichuan is located in the southwestern part of China, and is mostly covered by masses of high mountains. Its western part belongs to the Hengduan Mountains, one of the well-known biodiversity hotspots in the world (Myers et al. 2000). Eleven species of *Neopanorpa* have been recorded from Sichuan so far (Carpenter 1938, 1945; Cheng 1949, 1957; Wang and Hua 2017). However, the taxonomy of *Neopanorpa* is poorly known yet in this region to date. Recently, an eye-catching species of *Neopanorpa* from Sichuan with a greatly elongate notal organ and a pair of setiferous gonostyli was recognized as new to science. The discovery of this new species raises the number of *Neopanorpa* in Sichuan to twelve species in total. A key to Sichuan species of *Neopanorpa* is provided.

## Materials and methods

Adult specimens were captured in the mountainous area of Sichuan province, China in July 2016 and preserved in 75% ethanol. The holotype and paratypes are deposited in the Entomological Museum, Northwest A&F University, Yangling, China (**NWAFU**).

Males and females were dissected and photographs were taken with an advanced Stereo Microscope system Discovery V20 (Zeiss, Germany). Serial photographs were stacked with software Helicon Focus Pro 6.2.2 and further processed with Adobe Photoshop CS6. The measurements of wings were conducted with an electronic digital caliper.

## Results

### Key to Sichuan species of *Neopanorpa* (males)

Males of the following species are unknown: *N. banksi* Carpenter, *N. latipennis* Cheng, *N. parva* Carpenter, and *N. varia* Cheng.

**Table:** 

1	Notal organ extended beyond abdominal tergum VI	**2**
–	Notal organ extended not beyond abdominal tergum IV	**3**
2	Body generally black	***N. setigera* sp. n.**
–	Body generally yellowish brown	***N. choui* Cheng**
3	Wing markings with broad apical band and pterostigmal band	**4**
–	Wing markings generally absent	**5**
4	Abdominal segment VII–IX (A7–A9) dark brown	***N. heii* Cheng**
–	A7–A9 yellowish brown	***N. chelata* Carpenter**
5	Vein R_2_ trifurcated	**6**
–	Vein R_2_ bifurcated	**7**
6	Thorax generally brown; notal organ extended nearly to posterior margin of abdominal tergum IV, ended with a pointed apex	***N. validpennis* Cheng**
–	Thorax with a brown median band; notal organ extended not beyond the middle of abdominal tergum IV, ended with a truncated apex	***N. taoi* Cheng**
7	A7–A9 yellowish brown	***N. claripennis* Carpenter**
–	A7–A9 uniformly black	***N. nigritis* Carpenter**

### Key to Sichuan species of *Neopanorpa* (females)

Females of the following species are unknown: *N. taoi* Cheng and *N. validpennis* Cheng.

**Table:** 

1	Wings mostly unmarked	**2**
–	Wings with distinct apical or pterostigmal band	**3**
2	Thorax with a brown median band; A7–A9 yellowish brown	***N. claripennis* Carpenter**
–	Thorax black; A7–A9 black	***N. nigritis* Carpenter**
3	Thorax generally black or brown	**4**
–	Thorax with a brown median band	**5**
4	Wing markings with pterostigmal band forked posteriorly	***N. parva* Carpenter**
–	Wing markings with pterostigmal band reduced to a stripe on pterostigma and three discrete spots lining diagonally	***N. setigera* sp. n.**
5	Genital plate with axis not extended beyond main plate	**6**
–	Genital plate with axis extended beyond main plate for a short length	**8**
6	Main plate of female genital plate protruded laterally on each side	**7**
–	Main plate of female genital plate not protruded laterally on each side	***N. heii* Cheng**
7	Genital plate with main plate bearing two processes basally	***N. banksi* Carpenter**
–	Genital plate with main plate bearing no process basally	***N. varia* Cheng**
8	Posterior arms of female genital plate with V-shaped incisions apically	***N. choui* Cheng**
–	Posterior arms of female genital plate without V-shaped incision apically	**9**
9	Rostrum uniformly yellowish brown	***N. chelata* Carpenter**
–	Rostrum with a brown longitudinal stripe along clypeus	***N. latipennis* Cheng**

#### Neopanorpa setigera sp. n.

Taxon classificationAnimaliaMecopteraPanorpidae

http://zoobank.org/7205D935-75D9-4D2E-B633-227BB1DA5774

Figs 1 , 2

##### Type material.

**Holotype: CHINA: Sichuan**: ♂, Shimian County (29°1.23'N, 102°23.65'E), 2000–2200 m, 02 July 2016, leg. Gui-Lin Hu and Wei Du, ME000285 (NWAFU). **Paratypes.** 18♂♂16♀♀, same data as holotype, ME000286–ME000320 (NWAFU).

##### Diagnosis.

The new species can be readily recognized from its congeners by the following characters: body mostly black; wings slightly tinged with brown, wing markings only with greatly reduced apical band and a stripe on the pterostigma; notal process greatly elongated and extended beyond the posterior margin of abdominal tergum VI; postnotal organ well-developed, represented as three hirsute protruded areas on abdominal terga IV–VI respectively; male gonostylus bearing a cluster of long setae on central portion ventrally; female genital plate with a pair of parallel posterior arms slightly longer than axis.

##### Description of male

**Figure 1.**
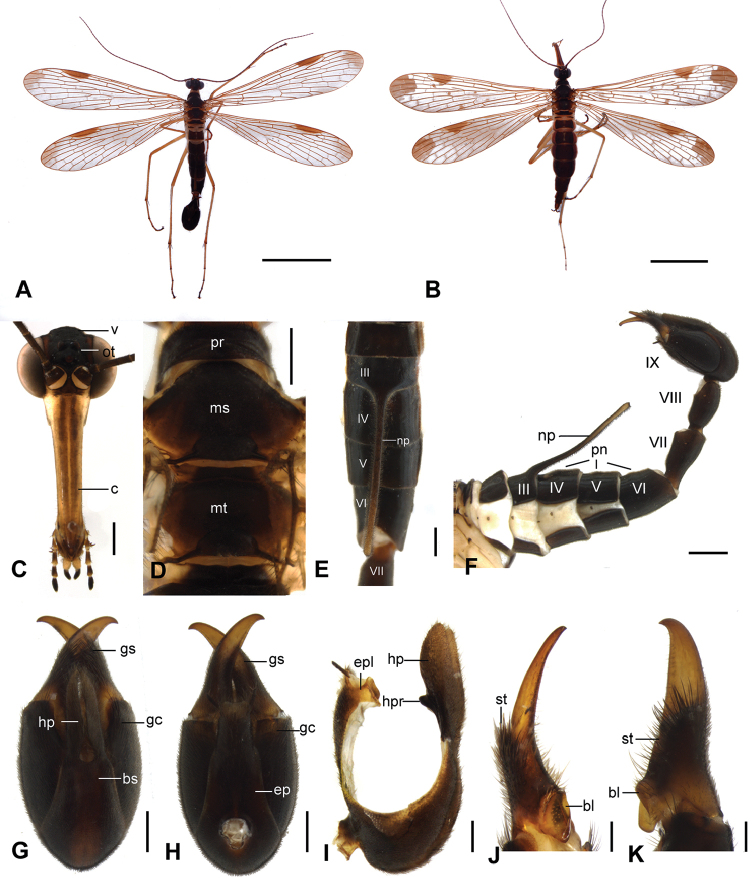
*Neopanorpa setigera* sp. n. **A** Male habitus, dorsal view **B** Female habitus, dorsal view **C** Head, frontal view **D** Thorax, dorsal view **E** Notal organ, dorsal view **F** Male abdomen, left lateral view **G** Male genital bulb, ventral view **H** Male genital bulb, dorsal view **I** Male 9th abdominal tergum and sternum, lateral view **J** Gonostylus, lateral view **K** Gonostylus, ventral view. Abbreviations: **bl** basal lobe **bs** basal stalk **c** clypeus **ep** epandrium **epl** epandrial lobe **gc** gonocoxite **gs** gonostylus **hp** hypovalve **hpr** hypandrial process **ms** mesoscutum **mt** metascutum **np** notal process **ot** ocellar triangle **pn** postnotal organ **pr** pronotum **st** setae **v** vertex. Scale bars: 5 mm (**A, B**); 0.5 mm (**C–E, G–H**); 0.2 mm (**I–K**); 1 mm (**F**).

(Fig. 1A). Forewing length 13.53 ± 0.40 mm, width 4.48 ± 0.18 mm; hindwing length 12.48 ± 0.38 mm, width 3.23 ± 0.10 mm (*n* = 10).

*Head* (Fig. 1C). Vertex and ocellar triangle black. Rostrum yellowish brown with two dark brown longitudinal stripes along clypeus.

*Thorax* (Fig. 1D). Pronotum black. Meso- and metascutum mostly blackish brown, grading to light brown laterally adjacent to wing base.

*Wings* (Fig. 1A). Slightly tinged with brown; R_2_ bifurcated. Wing markings dark brown, mostly absent, only with apical band reduced to obscure markings at apical part of wings and pterostigmal band reduced to a slender stripe on pterostigma.

*Abdomen*. Terga I–V black (Fig. 1A, E). Abdominal segment VI (A6) black, grading to brown at caudal end, A7–A8 blackish brown, constricted at base (Fig. 1F). Notal process on tergum III triangular at base, extending caudally as a long setiferous stick beyond the posterior margin of tergum VI; postnotal organ represented as three discontinuous hirsute protruded areas on terga IV–VI, respectively (Fig. 1F).

*Male genitalia* (Fig. 1G–H). Generally black. Hypandrium with broad basal stalk for nearly half length; basal stalk black on lateral areas and brown mesally; hypovalves slender, tapering toward membranous setiferous apices, extending to middle of gonostylus (Fig. 1G); in lateral aspect, hypovalves expanded dorsally at apical half into broad elliptical lobes, with conical hypandrial processes projected dorsally at basal third (Fig. 1I). Epandrium broad, tapering toward apex, with lateral margins abruptly narrowed at apical third, ended with truncate membranous apex; epandrial lobes subrectangular, yellowish brown (Fig. 1I). Gonostylus stout, strongly concave at outer margin near base (Fig. 1K), furnished with a cluster of black long setae ventrally in central part; median tooth barely raised; basal lobe large, flat, greatly concaved mesally (Fig. 1J, K).

**Figure 2.**
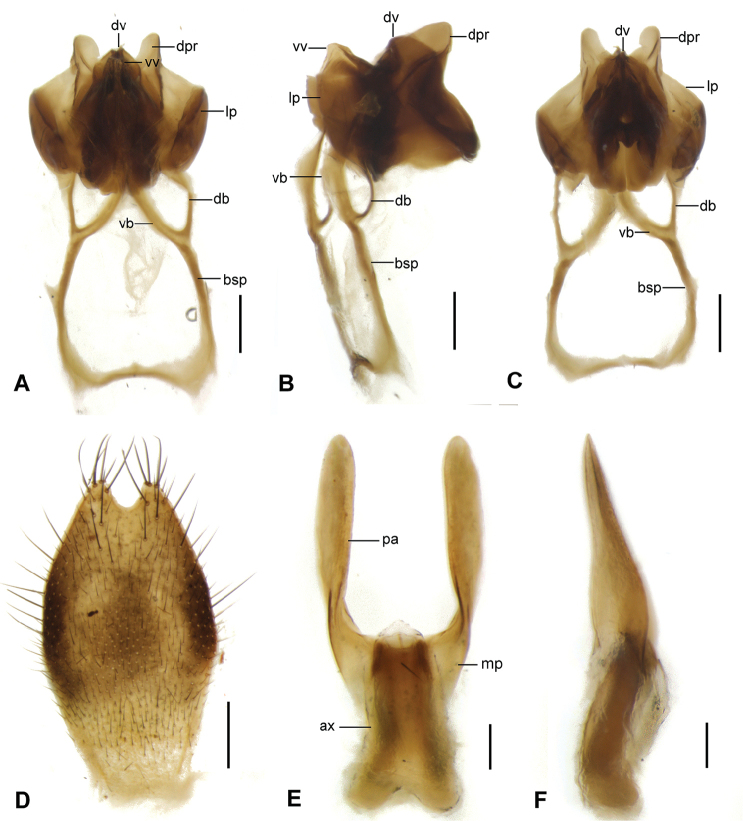
*Neopanorpa setigera* sp. n. **A** Aedeagal complex, ventral view **B** Aedeagal complex, lateral view **C** Aedeagal complex, dorsal view **D** Subgenital plate, ventral view **E** Genital plate, ventral view **F** Genital plate, lateral view. Abbreviations: **ax** axis **bsp** basal stalk of paramere **db** dorsal branch of paramere **dpr** dorsal process **dv** dorsal valve **lp** lateral process **mp** main plate **pa** posterior arm **vb** ventral branch of paramere **vv** ventral valve. Scale bars 0.2 mm (**A–D**); 0.1 mm (**E, F**).

*Aedeagal complex* (Fig. 2A–C). Strongly sclerotized. Ventral valves expanded ventrally into broad lobes, tapering toward apices; dorsal valves large, slightly longer than ventral valves (Fig. 2A, B). Paramere Y-shaped, with very long basal stalk; the stalks fused basally as large subrectangular frame; paramere forked distally into ventral branch and dorsal branch; ventral branch slender basally but broad-lobed, membranous apically and extended to the middle of ventral valves (Fig. 2A); dorsal branch slender, arc-shaped (Fig. 2B); lateral process greatly developed, broadly expanded in the same length with ventral valves, curved ventro-mesally, with apex rounded and projected distally; dorsal process large, auriculate (Fig. 2A–C).

##### Description of female

(Fig. 1B). Forewing length 14.43 ± 0.40 mm, width 3.34 ± 0.12 mm; hindwing length 13.28 ± 0.35 mm, width 3.18 ± 0.11 mm (*n* = 10). Same pattern as in the male. Female with more extensive wing markings. In forewings, apical band more extensive with pterostigmal band represented as a wide stripe at pterostigma and three little discrete spots lining diagonally. Abdomen black.

*Female genitalia.* Subgenital plate (Fig. 2D) long elliptical, with deep V-shaped emargination at apex, central part and median lateral margin blackish brown, the remainder yellowish brown, bearing long setae along margins. Genital plate (Fig. 2E, F) with axis extending anteriorly beyond main plate for half length, forked slightly proximally; posterior arms greatly developed, spatulate, extended caudally in parallel, slightly longer than axis, strongly constricted near base, rounded at apices; in lateral aspect, posterior arms greatly expanded ventrally at base and axis slightly curved dorsally.

##### Distribution.

China (Sichuan).

##### Etymology.

The specific epithet is derived from the Latin, *setigera* meaning setiferous, referring to the cluster of long dense setae on the male gonostyli.

##### Remarks.

*N. setigera* sp. n. resembles *N. nigritis* Carpenter, 1938 from Sichuan in black body and absence of wing markings. However, the extremely long notal organ of *N. setigera* sp. n. makes it easy to distinguish from *N. nigritis*. In addition, *N. choui* Cheng, 1949 in Sichuan has the similar lengthened notal organ. However, these two species differ greatly in body coloration. *N. choui* is yellowish brown, while *N. setigera* sp. n. is mostly black.

##### Habitat.

Adult specimens were captured on lower broad-leaved herbs or shaded vegetation under trees in lush evergreen forests. The environment is enclosed and moist.

## Discussion

*Neopanorpa setigera* sp. n. is similar to the Chinese species *N. choui*, *N. longiprocessa*, and *N. moganshanensis* Zhou & Wu by bearing an elongate notal organ extending beyond abdominal tergum VI. Compared with those species, the new species is unusual due to its greatly developed postnotal organ. The postnotal organ is barely raised, yet represented as a long stripe of hirsute area in *N. choui*, extending from the anterior margin to the posterior margin of abdominal tergum IV. Alternatively, the postnotal organs are both represented as a raised process with dense setae on abdominal tergum IV in *N. moganshanensis* and *N. longiprocessa* (Zhong and Hua 2013). The postnotal organ is limited on abdominal tergum IV in all the other species, but consists of three protruded areas with dense setae on abdominal terga IV–VI in *N. setigera* sp. n., respectively. *N. setigera* sp. n. displays the most developed postnotal organ in the extant species of Panorpidae.

The developmental degree of notal organ is closely related with mating behavior and implies different mating strategies (Thornhill 1980; Zhong et al. 2015b). The mating behavior of *N. longiprocessa* suggests that the highly developed notal process is a plausible sign of coercive mating because it can greatly reinforce the male control to the female wings (Zhong and Hua 2013). Therefore, we may assume that the greatly developed postnotal organ of *N. setigera* sp. n. also can increase the control to the female wings during copulation. In this case, the new species is likely to employ forced mating strategy without nuptial gifts during copulation, as in *N. longiprocessa*, although this needs to be confirmed in the future.

Another peculiar character of this new species is that it bears a cluster of black long setae on the central portion of male gonostylus ventrally. This feature is rarely found in Panorpidae. In *N. brisi* (Navás) and *N. effusa* (Navás), the male gonostylus only bears setae along the outer margin, but is glabrous on the ventral surface (Rust and Byers 1976). Although the male gonostylus is furnished with dense setae ventrally and along its outer margin in *N. pendula* Qian & Zhou, the setae are much shorter and less striking than those in *N. setigera* sp. n. Only the Indian species *N. hirsuta* (Crampton) has similarly prominent long dense setae on the gonostylus (Carpenter 1931b; Rust and Byers 1976). However, the gonostylus is furnished with black setae for nearly two-thirds in *N. hirsuta*, while is only furnished with dense setae on the central portion in *N. setigera* sp. n. Apart from the similar gonostyli, these two species differ greatly in other characters, such as wing markings, male genitalia, and female genital plate.

## Supplementary Material

XML Treatment for Neopanorpa setigera
